# Tauopathy in transgenic (SHR72) rats impairs function of central noradrenergic system and promotes neuroinflammation

**DOI:** 10.1186/s12974-016-0482-1

**Published:** 2016-01-20

**Authors:** Boris Mravec, Katarina Lejavova, Peter Vargovic, Katarina Ondicova, Lubica Horvathova, Petr Novak, Georg Manz, Peter Filipcik, Michal Novak, Richard Kvetnansky

**Affiliations:** Institute of Experimental Endocrinology, Slovak Academy of Sciences, Vlarska 3, 833 06 Bratislava, Slovakia; Institute of Physiology, Faculty of Medicine, Comenius University in Bratislava, Bratislava, Slovakia; Institute of Neuroimmunology, Slovak Academy of Sciences, Bratislava, Slovakia; Axon Neuroscience SE, Bratislava, Slovakia; LDN, Labor Diagnostika Nord, Nordhorn, Germany

**Keywords:** Alzheimer’s disease, Locus coeruleus, Neuroinflammation, Stress, Tauopathy, Transgenic rats

## Abstract

**Background:**

Brain norepinephrine (NE) plays an important role in the modulation of stress response and neuroinflammation. Recent studies indicate that in Alzheimer’s disease (AD), the tau neuropathology begins in the locus coeruleus (LC) which is the main source of brain NE. Therefore, we investigated the changes in brain NE system and also the immune status under basal and stress conditions in transgenic rats over-expressing the human truncated tau protein.

**Methods:**

Brainstem catecholaminergic cell groups (LC, A1, and A2) and forebrain subcortical (nucleus basalis of Meynert), hippocampal (cornu ammonis, dentate gyrus), and neocortical areas (frontal and temporal association cortices) were analyzed for NE and interleukin 6 (IL-6) mRNA levels in unstressed rats and also in rats exposed to single or repeated immobilization. Moreover, gene expression of NE-biosynthetic enzyme, tyrosine hydroxylase (TH), and several pro- and anti-inflammatory mediators were determined in the LC.

**Results:**

It was found that tauopathy reduced basal NE levels in forebrain areas, while the gene expression of IL-6 was increased in all selected areas at the same time. The differences between wild-type and transgenic rats in brain NE and IL-6 mRNA levels were observed in stressed animals as well. Tauopathy increased also the gene expression of TH in the LC. In addition, the LC exhibited exaggerated expression of pro- and anti-inflammatory mediators (IL-6, TNFα, inducible nitric oxide synthases 2 (iNOS2), and interleukin 10 (IL-10)) in transgenic rats suggesting that tauopathy affects also the immune background in LC. Positive correlation between NE and IL-6 mRNA levels in cornu ammonis in stressed transgenic animals indicated the reduction of anti-inflammatory effect of NE.

**Conclusions:**

Our data thus showed that tauopathy alters the functions of LC further leading to the reduction of NE levels and exaggeration of neuroinflammation in forebrain. These findings support the assumption that tau-related dysfunction of LC activates the vicious circle perpetuating neurodegeneration leading to the development of AD.

## Background

Sporadic Alzheimer’s disease (AD) represents the most prevalent form of dementia in the elderly, with several hypotheses being proposed to explain its etiopathogenesis. These hypotheses have focused either on the role of amyloid β, hyperphosphorylated and truncated tau protein, neuroinflammation, altered insulin signalization, impaired blood-brain barrier permeability, or other related factors and mechanisms [1, 2]. However, despite such enormous scientific effort, the primary factors responsible for the development of AD still remain only vaguely defined.

Recently accumulated evidence indicates that alteration of the brain’s noradrenergic system plays an important role in the development of AD-related neuropathology during the early stages of AD. Central norepinephrine (NE)-synthesizing neurons are located predominantly in the brainstem, particularly in the locus coeruleus (LC). These neurons richly ramify and innervate structures throughout the neuraxis, providing NE to all brain and spinal cord structures [3]. NE released from these neurons reduces neuroinflammation and potentiates synaptic plasticity and neurogenesis. In addition, NE modulates energy metabolism, the activity of astrocytes and microglia, cortical perfusion, and permeability of the blood-brain barrier [4–7]; all functions impaired in AD patients. Moreover, recent findings indicate that tau pathology initially occurs in specific brainstem nuclei, particularly the LC, and spreads from these nuclei to the forebrain [8–10]. Therefore, it has been suggested that the LC may represent the center of AD-related neuropathology [11].

Based on the abovementioned facts, we investigated the interrelations between brain NE and markers of neuroinflammation in discrete brain areas of both wild-type as well as in transgenic (SHR72) rats expressing a pathological form of tau protein at basal conditions. However, since brain NE-synthesizing neurons are activated during stress, which is implicated in the development of AD [12–15], we also investigated the effect of both single and repeated immobilization (IMO) stress on NE and interleukin 6 (IL-6) mRNA levels in brain structures known to be involved in AD neuropathology. In our study, we focused on brainstem catecholaminergic cell groups (LC, A1, A2) as well as the forebrain structures innervated by the LC, including cornu ammonis (CA), dentate gyrus (DG), nucleus basalis of the Meynert (NBM), and both frontal (FCx) and temporal (TCx) association cortices. In addition, in the LC, we analyzed gene expression of the NE-biosynthetic enzyme, and tyrosine hydroxylase (TH), as well as several pro- and anti-inflammatory mediators.

## Methods

### Animals

Experiments were performed on 6-month-old male transgenic (SHR72) and age-matched wild-type rats of the same SHR genetic background. The transgenic (SHR72) animals expressed a fragment of tau protein containing four C-terminal repeats designed to correspond with the pathological tau protein found in human AD brains. The transgenic (SHR72) animals were prepared by microinjection of human cDNA encoding N- and C-terminal truncated tau protein encompassing the region of amino acids 151–391 into the male pronucleus of 1-day-old rat embryos. The transgenic construct (human truncated MAPT cDNA under Thy-1 promoter) was devoid of all prokaryotic sequences before injection. The injected embryos were incubated in vitro and implanted to the oviducts of pseudo-pregnant foster mothers about 12 h after the injection. This animal model recapitulates basic features of human neurofibrillary pathology typical of AD sufferers and is the unique transgenic model that unequivocally demonstrates that expression of pathologically truncated tau protein is sufficient to drive neuropathology typical of AD [16–18].

The homozygous transgenic (SHR72) animals with eight copies of the transgene per genome only survived to the age of 4 months. Therefore, we bred hemizygous transgenic (SHR72) animals with wild-type animals of the same genetic background, which expanded the lifespan of transgenic (SHR72) animals to an average of 7 months. The transgene was transmitted to subsequent generations according to Mendelian law, and the colony was phenotypically stable at the time the animals used in this study were taken (12th generation).

The animals, bred at the Institute of Neuroimmunology, were kept under controlled conditions in the animal facility (12-h light/12-h dark cycle, lights on/off at 0600 hours; temperature, 22 ± 1 °C) with free access to tap water and standard pelleted rat chow. All experiments were performed between 0800 and 1200 hours, with all external noises or any other stressful stimuli of the animals being strictly avoided. The experiments were carried out in accordance with the Council Directive 2010/63EU of the European Parliament and the Council of 22 September 2010 on the protection of animals used for scientific purposes.

### Immobilization stress

**Fig. 1:**
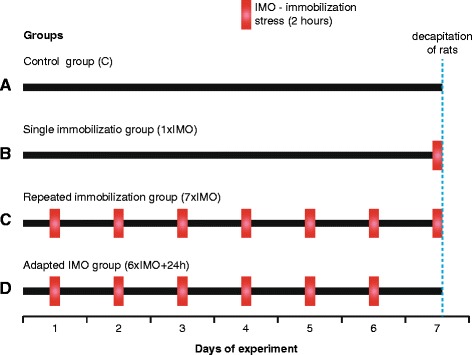
Schematic illustration of experimental design. **a** Control group. **b** Single immobilization group (1×IMO). **c** Repeated immobilization group (7×IMO). **d** Adapted IMO group (6×IMO+24 h)

In the experiments, we used unstressed (control) and stressed groups of wild-type and transgenic (SHR72) rats (Fig. 1):

- Control groups (C): unstressed animals (*n* = 9 of both wild-type and transgenic rats) that were decapitated immediately after being taken out from their home cages.
- Single IMO group (1×IMO): the animals (*n* = 9 of both wild-type and transgenic rats) that were subjected once to 2 h IMO and were decapitated immediately after the end of stressor exposure.
- Repeated IMO group (7×IMO): the animals (*n* = 10 of both wild-type and transgenic rats) were subjected repeatedly to IMO for 2 h daily for seven successive days and decapitated immediately after the last IMO was finished.
- Adapted IMO group (6×IMO + 24 h): the animals (*n* = 10 of both wild-type and transgenic rats) were subjected to IMO for 2 h daily for six successive days and decapitated 24 h after the last IMO, at the time when the seventh IMO would have been finished. This group enables to determine the effect of seventh exposure to IMO in repeatedly stressed rats and therefore to investigate the mechanisms of adaptation to chronic stress. The IMO stress was performed as previously described [19].

### Microdissection of brain areas

**Fig. 2:**
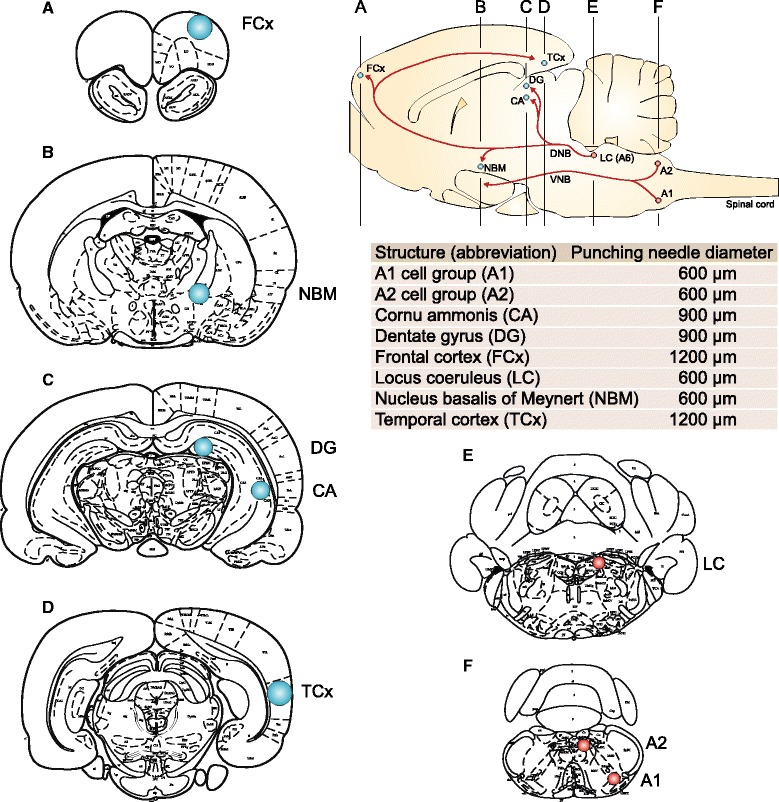
Localization of brainstem catecholaminergic cell groups (LC, A1, and A2) and forebrain areas in the rat brain (**a**-**f**) and diameters of punching needles used for their microdissections. Axons of A1 and A2 noradrenergic cell groups are a part of the ascending ventral noradrenergic bundle (VNB) innervating hypothalamus. Axons of the locus coeruleus (LC/A6) cell group constitute the ascending dorsal noradrenergic bundle (DNB) providing noradrenergic innervation of hippocampal cornu ammonis (CA) and dentate gyrus (DG), nucleus basalis of the Meynert (NBM), as well as frontal (FCx) and temporal cortex (TCx). *Red circles* norepinephrine synthesizing cell groups. *Blue circles* forebrain structures innervated by the locus coeruleus. Modified according to [23, 48, 49]

The tissue of brainstem catecholaminergic cell groups as well as that of the forebrain subcortical and cortical areas (Fig. 2) was dissected bilaterally by the micropunch technique [20] according to the original punching guide atlas [21]. Briefly, after the quick removal, the brains were immediately frozen on dry ice and cut into 300-μm-thick serial coronal sections using a cryostat (Reichert-Jung) at −10 °C. The tissue was isolated under a dissection microscope by specialized metal punching needles with a diameter ranging from 600 to 1200 μm (for details, see Fig. 2) and then stored at −70 °C for the later analyses.

### Determination of catecholamine in brain areas

Tissues of isolated brain areas were sonicated in 100 μL of 0.01 M HCl. Two microliters of homogenate were used for the determination of proteins using a bicinchoninic acid (BCA) protein assay (Thermo Scientific, Rockford, IL.). The appropriate amount of supernatant (containing 10–400 μg of protein, depending on brain area) was used for norepinephrine measurement using 3-CAT Research RIA kits (Labor Diagnostica Nord, Nordhorn, Germany) according to the manufacturer’s protocol. We have tested this method for different brain areas, and we obtained a good linearity of measurements between 10 and 800 pg of NE or 10 and 400 μg protein in homogenates. The final loading amounts of homogenates were LC and NBM, 10–20 μg of protein; A1 and A2 noradrenergic cell groups, 20–30 μg of protein; temporal cortex, 100–200 μg of protein; frontal cortex, 300–400 μg of protein; and CA, 300–400 μg of protein. The sensitivity of the RIA assay for NE in the brain tissue was 7.2 pg/sample making it sensitive enough for the performed experiments. The values were normalized to proteins determined in the homogenate and expressed as nanograms of catecholamine per milligram of protein.

### Isolation of RNA and measurement of mRNA levels by RT-PCR

Total RNA was isolated from the frozen tissue samples of the brain areas using TRI REAGENT (MRC, Inc.). Reverse transcription was performed from total RNA using Ilustra™ Ready-To-Go™ RT-PCR beads (GE Healthcare, Buckinghamshire, UK) with pd(N)_6_ primer according to manufacturer’s protocol.

Gene expression was determined by RT-PCR analyses. For all PCR amplifications, cDNA aliquots containing 20 ng of total RNA were used. Transcripts were amplified with specific primers (shown in Table 1) using a SensiFast Hi-ROX qPCR Kit (Bioline, Taunton, MA) according to the manufacturer’s instructions in 20 μL of total reaction volume using an ABI Prism 7900HT Sequence Detection System thermocycler (Applied Biosystems, Inc, Foster City, CA, USA). Data were analyzed with sodium dodecyl sulfate (SDS) software version 2.3 (Applied Biosystems) and inspected to determine artifacts (loading errors, threshold errors, etc.). Count numbers (Ct values) were exported to an Excel spreadsheet and analyzed according to the ΔΔCT method described by Livak and Schmittgen [22]. For internal controls, several genes were tested including 18S rRNA, beta tubulin, GAPDH, TATA-binding protein, RPS21, and beta actin. We normalized the results of the investigated genes to these internal controls and found similar results regarding both gene expression changes and patterns. Subsequently, we examined the variability of values between samples within experimental groups and concluded that 18S rRNA would be the most appropriate internal control.

**Table 1:** Sequences of primers used for amplification of target cDNA of interleukin 6 (IL-6), tyrosine hydroxylase (TH), tumor necrosis factor alpha (TNF-α), inducible nitric oxide synthases 2 (iNOS2), interleukin 10 (IL-10), transforming growth factor beta 1 (TGFβ1), and 18S ribosomal protein

Gene	Primer	Oligonucleotide sequence
IL-6	Forward	AAGTCGGAGGCTTAATTACATATGTTC
	Reverse	TCATCGCTGTTCATACAATCAGAA
TH	Forward	TCTCCCTGAGGGGTACAAAA
	Reverse	GAATTTTGGCTTCAAATGTCTCA
TNF-α	Forward	TGATCGGTCCCAACAAGGA
	Reverse	TGGGCTACGGGCTTGTCA
iNOS2	Forward	GCTTCAGAATGGGGAGCTG
	Reverse	AGGTTGGAGGCCTTGTGTC
IL-10	Forward	CCCTGGGAGAGAAGCTGAAGA
	Reverse	CACTGCCTTGCTTTTATTCTCACA
TGFβ1	Forward	CCTGGAAAGGGCTCAACAC
	Reverse	CAGTTCTTCTCTGTGGAGCTGA
18S	Forward	ATGGTTCCTTTGTCGCTCGCTCC
	Reverse	TGGATGTGGTAGCCGTTTCTCAGG

### Western blot analysis

For detection of TH protein, freshly frozen brain samples were homogenized in ice-cold extraction buffer (20 mM Tris-HCl, pH 8, 100 mM NaCl, 1 mM EDTA, 1 mM dithiothreitol, 0.5 % Triton X-100, 20 mM NaF, and 1 mM activated Na_3_VO_4_) supplemented with protease inhibitor cocktail (Complete without EDTA; Roche, Manheim, Germany) using the disposable polypropylene homogenizers fitted to the tip of 1.5 mL eppendorf tube (E&K Scientific Products, CA, USA). The samples were centrifuged for 20 min at 16,000×*g*, and supernatants were transferred into new tubes. Concentrations of total proteins were measured by PierceTM BCA protein assay kit (Thermo Scientific, USA). Samples were mixed with standard 2× sample loading buffer (10 mM Tris-HCl, 4 % SDS, 4 % 2-mercaptoethanol, 20 % glycerol, and 0.1 % Coomassie Brilliant Blue G-250) and heated at 95 °C for 5 min. Twenty micrograms of total protein was then separated by electrophoresis in 12 % SDS-polyacrylamide gels. Separated proteins were transferred onto nitrocellulose membranes (Whatman, Maidstone, UK), which were blocked in Odyssey blocking buffer (LI-COR, Lincoln, NE, USA) containing 0.1 % Tween 20 (Sigma, St. Louis, MO, USA). Membranes were incubated overnight with primary monoclonal antibody (anti-TH, MAB5280, Millipore, dilution 1:3000) in Odyssey blocking buffer (LI-COR, Lincoln, NE, USA) containing 0.1 % Tween 20 (Sigma, St. Louis, MO, USA). Secondary infrared 800 CW goat anti-mouse IgG (LI-COR, Lincoln, NE, USA) was diluted 1:10,000 in Odyssey blocking buffer (LI-COR, Lincoln, NE, USA) containing 0.1 % Tween 20 (Sigma, St. Louis, MO, USA) and incubated with membrane for 2 h at RT. The blots were scanned with Odyssey infrared imaging system (LI-COR, Lincoln, NE, USA). Detected bands were quantified using Image Studio software ver. 2.0.38 (LI-COR, Lincoln, NE, USA).

#### Statistical analysis

**Fig. 3:**
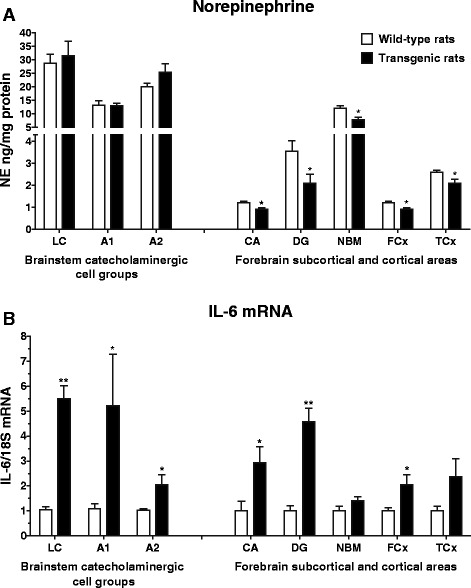
Basal levels of norepinephrine (NE) and interleukine-6 mRNA (IL-6 mRNA) in brainstem catecholaminergic cell groups containing NE cell bodies (LC, A1, A2) and forebrain subcortical and cortical areas in wild-type (*white square*) and transgenic (SHR72) rats (*black square*). Each value is the mean ± SEM (*n* = 4–10). Statistical significance vs. corresponding wild-type group: *P < 0.05; **P < 0.01

**Fig. 4:**
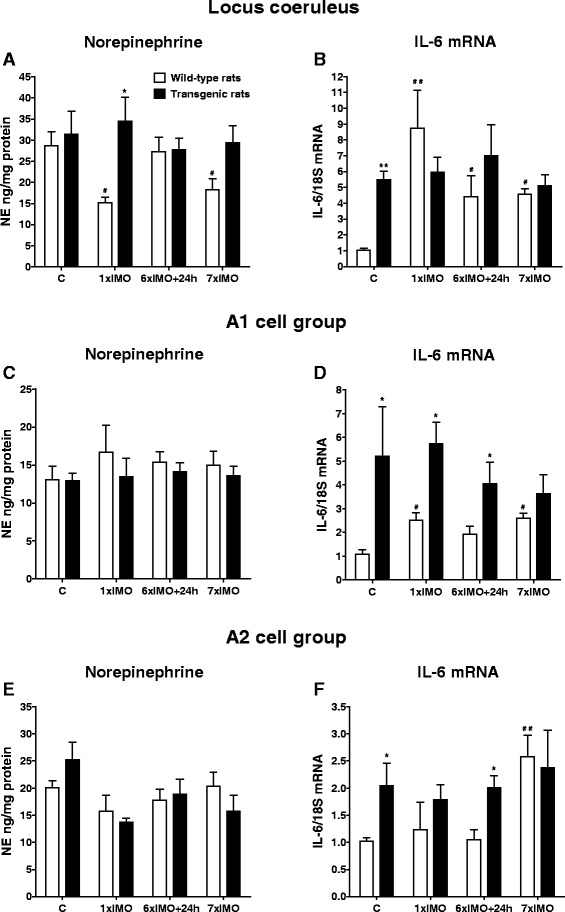
Levels of norepinephrine (NE) and interleukine-6 mRNA (IL-6 mRNA) in the LC, A1, and A2 brainstem noradrenergic areas in wild-type (*white square*) and transgenic (SHR72) rats (*black square*) exposed to a single (1 × 2 h) or repeated (7 × 2 h) immobilization stress (IMO). *C* control rats; 6×IMO + 24 h-adapted IMO rats exposed to 6×IMO for 2 h and decapitated 24 h after the last IMO. Each value is the mean ± SEM (*n* = 5–8). Statistical significance vs. corresponding wild-type group: *P < 0.05; **P < 0.01; statistical significance vs. wild-type control group: ^#^ *P* < 0.05; ^##^ *P* < 0.01

**Fig. 5:**
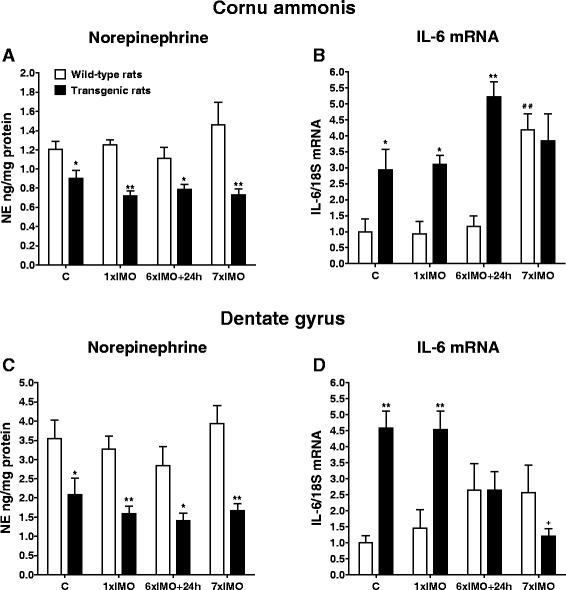
Levels of norepinephrine (NE) and interleukine-6 mRNA (IL-6 mRNA) in the cornu ammonis (CA) and dentate gyrus (DG) in wild-type (*white square*) and transgenic (SHR72) rats (*black square*) exposed to a single (1 × 2 h) or repeated (7 × 2 h) immobilization stress (IMO). *C* control; 6×IMO + 24 h-adapted IMO rats exposed to 6×IMO for 2 h and decapitated 24 h after the last IMO. Each value is the mean ± SEM (*n* = 5–8). Statistical significance vs*.* corresponding wild-type group: *P < 0.05; **P < 0.01; statistical significance vs. wild-type control group: ^#^ *P* < 0.05; ^##^ *P* < 0.01; statistical significance vs. transgenic control group: ^+^ *P* < 0.05

**Fig. 6:**
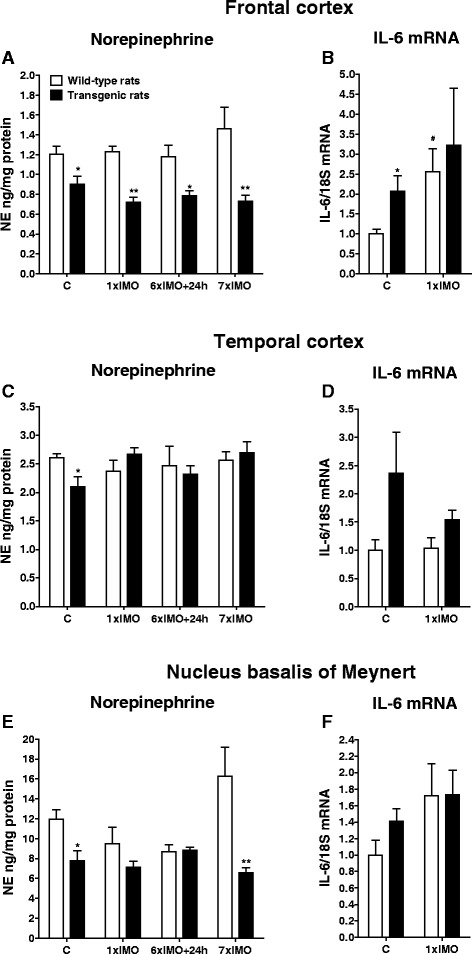
Levels of norepinephrine (NE) and interleukine-6 mRNA (IL-6 mRNA) in frontal cortex, temporal cortex, and nucleus basalis of Meynert in wild-type (*white square*) and transgenic (SHR72) rats (*black square*) exposed to a single (1 × 2 h) or repeated (7 × 2 h) immobilization stress (IMO). *C* control; 6×IMO + 24 h-adapted IMO rats exposed to 6×IMO for 2 h and decapitated 24 h after the last IMO. Each value is the mean ± SEM (*n* = 4–10). Statistical significance vs. corresponding wild-type group: *P < 0.05; **P < 0.01; statistical significance vs*.* wild-type control group: ^#^ *P* < 0.05

**Fig. 7:**
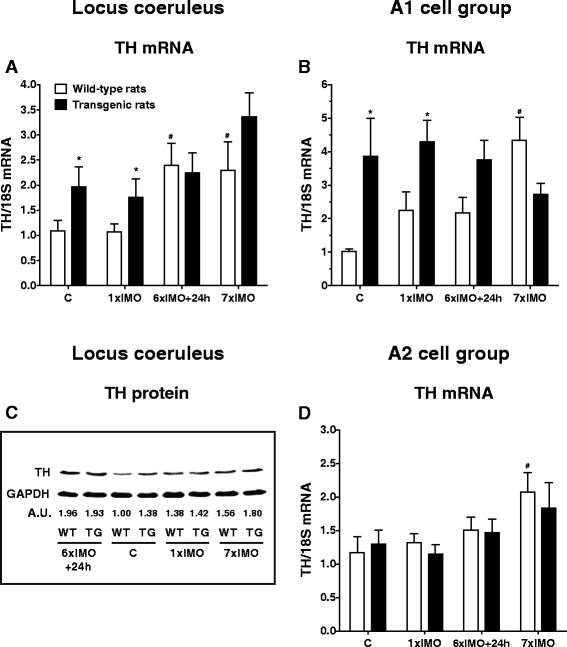
Gene expression (**a**, **b**, and **d**) and protein (**c**) levels of tyrosine hydroxylase (TH) in the LC, A1, and A2 brainstem noradrenergic cell groups of wild-type (*white square*) and transgenic (SHR72) rats (*black square*) exposed to a single (1 × 2 h) or repeated (7 × 2 h) immobilization stress (IMO). Each sample of TH protein represents a pool of two to three animals (**c**), values are expressed as arbitrary units (A.U.) calculated as a ratio between TH and GAPDH in a given sample. *C* control; 6×IMO + 24 h-adapted IMO rats exposed to 6×IMO for 2 h and decapitated 24 h after the last IMO. Each value is the mean ± SEM (*n* = 5–8; **a**, **b**, and **d**). Statistical significance vs. corresponding wild-type group: *P < 0.05; statistical significance vs. wild-type control group: ^#^ *P* < 0.05

**Fig. 8:**
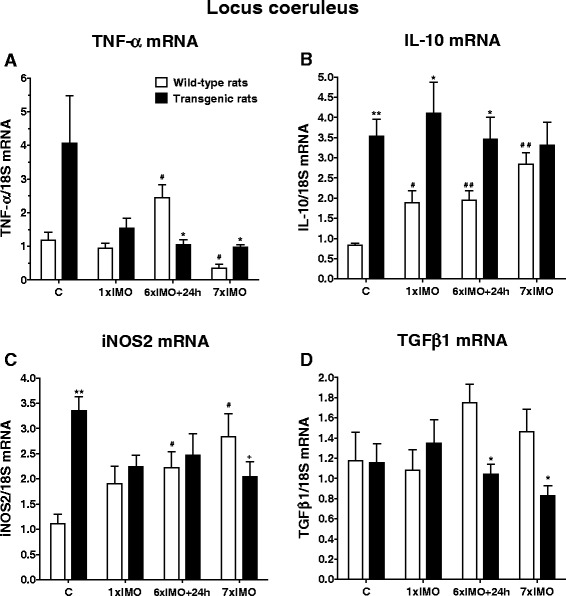
Gene expression of pro-inflammatory factors TNF-α (**a**), iNOS2 (**c**), and anti-inflammatory factors IL-10 (**b**) and TGFβ1 (**d**) in the LC of wild-type (*white square*) and transgenic (SHR72) rats (*black square*) exposed to a single (1 × 2 h) or repeated (7 × 2 h) immobilization stress (IMO). *C* control; 6×IMO + 24 h-adapted IMO rats exposed to 6×IMO for 2 h and decapitated 24 h after the last IMO. Each value is the mean ± SEM (*n* = 5–8). Statistical significance vs. corresponding wild-type group: *P < 0.05; **P < 0.01; statistical significance vs. wild-type control group: ^#^ *P* < 0.05, ^##^ *P* < 0.01

**Fig. 9:**
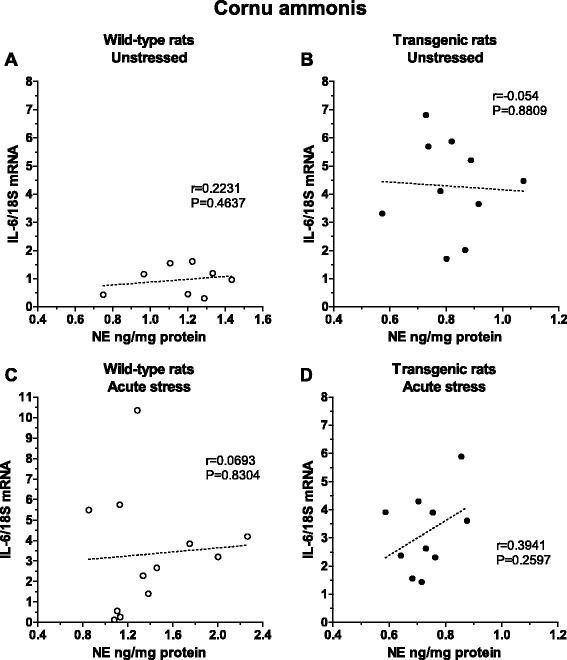
Correlation plots illustrating the relationship of norepinephrine (NE) concentration and interleukine-6 mRNA (IL-6 mRNA) in cornu ammonis (CA) of wild-type controls (**a**, *white circle*), transgenic controls (**b**, *black circle*), wild-type rats exposed to a single 2-h immobilization stress (**c**, *white circle*), and transgenic rats exposed to a single 2-h immobilization stress (**d**, *black circle*)

All statistical analyses were performed using SigmaPlot 11.0 (Systat Software, Inc., Germany). The normal distribution of the quantitative data was verified for all analyzed data using the Kolmogorov-Smirnov test allowing for parametric tests to be applied. Statistical analysis of the data was performed by *t* test (used for the comparison of changes in NE and IL-6 mRNA levels between unstressed wild-type and transgenic rats; Fig. 3) and by one-way ANOVA measurement followed by a Dunnett’s test (used for the comparison of changes in NE levels as well as in mRNA levels of TH and cytokines in rats exposed to a single or repeated immobilization; Figs. 4, 5, 6, 7, and 8). The correlation between NE levels and IL-6 mRNA levels was determined by linear regression test (Fig. 9). The results are expressed as means ± SEM and represent an average of four to ten animals. A *P* value of <0.05 was taken as indicative of statistical significance for the tests.

## Results

### Effect of tauopathy on basal norepinephrine and IL-6 mRNA levels in brainstem and forebrain areas

Tauopathy significantly affected basal levels of NE as well as gene expression of IL-6, one of the major pro-inflammatory cytokines in the brain (Fig. 3). Although NE levels were not considerably changed in the brainstem catecholaminergic cell groups, they were clearly decreased in all forebrain structures innervated by the LC, particularly in hippocampal areas such as the CA and DG, as well as NBM, FCx, and TCx in transgenic (SHR72) animals compared to wild-type rats (*P* < 0.05; Fig. 3a). On the other hand, basal levels of IL-6 mRNA were elevated in all abovementioned investigated areas, the most prominent elevation being observed in the LC and DG of transgenic rats (*P* < 0.01; Fig. 3b).

These data led us to further study the question of whether the tauopathy itself affects NE and IL6 mRNA levels in the investigated brain areas of transgenic rats under the conditions of single and repeated stress by immobilization.

### Effect of tauopathy on stress-induced changes of norepinephrine levels in brainstem and forebrain areas

We found that in stressed rats, there were differences in NE levels within selected brain structures between the wild-type and transgenic groups. In general, in stressed transgenic rats, the concentrations of NE remained unchanged compared to their unstressed, transgenic controls.

In the LC, a significant reduction of NE concentration was found in wild-type animals exposed to either single or repeated IMO (*P* < 0.05; Fig. 4a). However, transgenic animals did not show this stress-induced reduction in NE levels (Fig. 4a). Furthermore, we did not find any significant changes in NE concentrations within the A1 noradrenergic cell group when examining singly or repeatedly stressed wild-type and transgenic rats (Fig. 4c). But, the A2 noradrenergic cells group showed a non-significant reduction in NE levels in both wild-type and transgenic animals exposed to single IMO (Fig. 4e).

We found that NE levels were reduced in both hippocampal regions (CA and DG) compared to wild-type animals in both the single (*P* < 0.01) and repeatedly stressed (*P* < 0.01) transgenic rats as well as in transgenic rats from the adapted IMO group (*P* < 0.05). Whereas repeated IMO non-significantly increased NE levels in the CA and DG of wild-type rats the NE levels remained unchanged or were even non-significantly reduced in transgenic rats when compared to controls (Fig. 5a, c).

Although we have seen significantly reduced NE levels in the FCx of transgenic compared to wild-type rats (*P* < 0.01, single and repeatedly stressed rats; *P* < 0.05, adapted IMO rats; Fig. 6a), we found no difference in the levels of NE levels in the TCx between stressed transgenic and wild-type rats (Fig. 6c). Whereas repeated stress non-significantly increased NE levels in the FCx of wild-type rats, NE levels did not change or were even non-significantly reduced in transgenic rats when compared to controls. Additionally, NE levels in the TCx were unchanged, while reduced NE levels were found in the entorhinal cortex (data not shown).

Repeated stress non-significantly increased NE levels in the NBM of wild-type rats. In transgenic rats, NE levels remained unchanged when compared to those of unstressed transgenic animals, but they were significantly lower than these in repeatedly stressed wild-type rats (*P* < 0.01; Fig. 6e).

### Effect of tauopathy on stress-induced changes of IL-6 mRNA levels in brainstem and forebrain areas

Both, single as well as repeated exposure to IMO stress induced a significant elevation of IL-6 mRNA level in the LC (*P* < 0.01, single stress; *P* < 0.05; adapted IMO and repeated stress; Fig. 4b) and A1 noradrenergic cell group (*P* < 0.05 single and repeated stress; Fig. 4d) of wild-type animals, However, IL-6 mRNA levels were only elevated in wild-type animals by repeated stress in the A2 noradrenergic cell group (*P* < 0.01; Fig. 4f), CA (*P* < 0.01; Fig. 5b), and FCx (*P* < 0.05; Fig. 6b).

Similar to the abovementioned findings of NE levels in stressed transgenic rats, exposure of rats to IMO did not induce further increases in IL-6 gene expression in selected brain areas of transgenic animals when compared to values found in unstressed transgenic animals (Figs. 4, 5, and 6). However, in some brainstem and in hippocampal areas, IL-6 mRNA levels were significantly elevated in stressed transgenic rats compared to corresponding wild-type groups (Figs. 4d, e, and 5b, d).

### Effect of tauopathy on basal and stress-induced changes of TH gene expression and TH protein levels in brainstem noradrenergic cell groups

Brainstem noradrenergic neurons, particularly these in the LC, provide NE to all levels of the neuraxis. Reduced NE levels and increased levels of IL-6 mRNA in investigated forebrain structures (Fig. 3a, b) indicate altered function of brainstem noradrenergic neurons. Therefore, when attempting to assess the functionality of the LC, we selected TH mRNA and protein levels as markers of NE synthesis. We also investigated the effect of stress on gene expression and protein levels of TH.

Repeated IMO significantly increased TH mRNA levels in LC of wild-type rats (*P* < 0.05; Fig. 7a). This is not surprising as TH gene expression in the LC of transgenic rats was already significantly increased in unstressed (*P* < 0.05) as well as in singly stressed transgenic rats when compared to corresponding wild-type animals (*P* < 0.05; Fig. 7a). Also, non-significantly increased TH mRNA levels were observed in repeatedly stressed transgenic rats (Fig. 7a). These stress-induced increases of TH in transgenic rats were confirmed by similar results found in TH protein levels (Fig. 7c).

We also investigated TH gene expression in the A1 and A2 noradrenergic cell groups. We found that TH mRNA levels in the A1 noradrenergic cell group of wild-type animals were significantly elevated only by repeated IMO stress (*P* < 0.05; Fig. 7b). When compared to wild-type animals, transgenic rats showed elevated basal levels (*P* < 0.05), but no further elevation was detected after either single or repeated stress (Fig. 7b). Similarly to the A1 noradrenergic cell group, gene expression of TH in the A2 noradrenergic cell group of wild-type animals was significantly elevated only by repeated IMO stress (*P* < 0.05; Fig. 7d).

In transgenic rats, stress did not induce any significant changes in TH mRNA levels. Similarly, no changes were seen when comparing the values of TH mRNA levels in the A2 noradrenergic cell groups between transgenic rats and corresponding wild-type animals (Fig. 7d).

### Effect of tauopathy on basal and stress-induced changes of levels of pro- and anti-inflammatory factors in the locus coeruleus

In transgenic rats, tauopathy is localized mainly to the brainstem [16]. Because tauopathy also induces neuroinflammation, we also investigated levels of selected pro- and anti-inflammatory factors as markers of the immune milieu of the LC.

Besides IL-6 mRNA, we determined in the LC expression of genes of other inflammatory factors, particularly the tumor necrosis factor alpha (TNF-α), inducible nitric oxide synthases 2 (iNOS2), interleukin 10 (IL-10), and transforming growth factor beta 1 (TGFβ1). Whereas mRNA levels of the pro-inflammatory factor TNF-α were only non-significantly elevated in unstressed transgenic rats (*P* = 0.078; Fig. 8a), the expression of the iNOS2 gene showed significant increases in the LC of transgenic animals during basal conditions (*P* < 0.01; Fig. 8c). However, after the exposure to repeated stress, TNF-α mRNA levels were reduced compared to corresponding wild-type rats (*P* < 0.05; Fig. 8a), while gene expression of iNOS2 was significantly reduced in repeatedly stressed transgenic rats when compared to unstressed transgenic rats (*P* < 0.05; Fig. 8c).

Gene expression of the anti-inflammatory cytokine IL-10 was significantly elevated during basal conditions in the LC of transgenic rats (*P* < 0.01; Fig. 8b). Even if stress had no effect on IL-10 mRNA levels in transgenic rats, gene expression of IL-10 was significantly elevated in both singly stressed and adapted IMO transgenic rats when compared to values found in corresponding wild-type animals (*P* < 0.05; Fig. 8b).

Gene expression of neuroprotective factor TGFβ1 in wild-type animals was not affected by stress. However, TGFβ1 mRNA levels were significantly lower in repeatedly stressed and adapted IMO transgenic rats compared to levels found in the corresponding wild-type animals (*P* < 0.05; Fig. 8d).

### Effect of tauopathy on correlation between norepinephrine and IL-6 mRNA levels in cornu ammonis under basal and stress conditions

The reduced NE and elevated IL-6 mRNA levels found in unstressed transgenic rats (Fig. 3), differences in NE and IL-6 mRNA levels in stressed transgenic animals (Figs. 4, 5, and 6), and altered immune background in the LC (Fig. 8) indicate the effect of deregulated immunomodulation on the brain’s noradrenergic system. Therefore, we investigated the effect of stress in both wild-type and transgenic rats on the relationship between NE and IL-6 mRNA levels in the CA, a brain structure innervated almost exclusively by the LC.

We did not find any correlation between NE and IL-6 mRNA levels in the CA of either unstressed or stressed wild-type (Fig. 9a, c) or transgenic rats (Fig. 9b, d). However, whereas NE levels are similar between unstressed wild-type and transgenic rats (ranging from 0.7 to 1.5 ng/mg of protein), the mean gene expression of IL-6 is almost three times higher in transgenic rats (Fig. 9a, b).

## Discussion

The brain’s noradrenergic system regulates the central stress response [23] and maintains several cognitive, affective, and behavioral functions [3, 24], while contributing to the consolidation of learning and memory [25]. However, Gibbs and Summers showed that NE released by brainstem noradrenergic neurons, besides its neurotransmitter role, is also involved in maintaining the brain’s tissue milieu [4]. Furthermore, centrally released NE modulates synaptic plasticity, neurogenesis, energy metabolism, activity of astrocytes and microglia, cortical perfusion, and the permeability of the blood-brain barrier [4–7]. Moreover, it also exerts several potent anti-inflammatory and anti-oxidative effects on the brain tissue [26]. All of the abovementioned “homeostatic” processes modulated by NE are impaired in the brains of AD patients. Importantly, recent findings of Braak et al. [9] indicate that in humans, pathological tau species are originally formed in the LC (the brainstem structure representing the main accumulation of noradrenergic neurons in the brain) and then spread by axonal transport and interneuronal communications to the transentorhinal region, then to the entorhinal region, the hippocampal formation, and then later to the association cortices [9]. Therefore, it is suggested that the LC plays a crucial role in the development of AD-related neuropathology [11]. The importance of the LC in AD development is further supported by the finding that LC destruction results in the worsening of neuroinflammation and amyloid β deposition in brains of transgenic Aβ animal models, as well as in elevated IL-1β and Ccl2 cytokines in laboratory animals [27, 28]. Interestingly, monoaminergic deficiency and severe depletion of NE has been found in most LC projection areas in AD patients [29]. Morphological studies of brains of AD patients have shown that the LC undergoes significant degeneration that precedes pathological changes in the forebrain [30]. It is suggested that this degeneration starts early in the course of AD pathogenesis and that the LC represents the centre from which altered tau proteins spread throughout the forebrain structures [9]. Therefore, we investigated the effect of tauopathy on the LC by using transgenic (SHR72) rats that over-express human truncated tau protein [17].

Preclinical investigation of the LC role in AD pathogenesis is based mainly on the study of the effects resulting from the destruction of this noradrenergic cell group on the development of AD-related neuropathology. However, in the available literature, information on tauopathy’s effect on LC functions and on the immune background of brain tissue during basal and stress conditions is lacking.

Based on the abovementioned facts, we investigated the mutual relationship between the brain’s noradrenergic system and immune activity in unstressed and singly or repeatedly stressed wild-type and transgenic rats that over-express human truncated tau protein [17]. Here, we demonstrate for the first time that expression of human truncated tau protein, whose primary sequence was derived from paired helical filaments obtained from the brains of AD patients, is sufficient to induce dysfunction of LC neurons in vivo and this dysfunction is most likely followed by exaggerated synthesis of pro-inflammatory cytokines in brain structures innervated by the LC that are known to be affected by neuropathology in AD patients.

We have found significant reduction of basal NE levels in hippocampus (CA and DG) and NBM as well as in cortical areas (frontal and temporal association cortex) of transgenic rats, structures innervated almost entirely by noradrenergic LC neurons [3]. Our findings indicate that the function of the noradrenergic system in the brains of transgenic rats is significantly impaired, at least at the level of NE synthesis. This is in agreement with observed significant reductions of NE levels on the cerebrospinal fluid of AD patients compared to matched controls [31].

Recent studies have revealed that NE exerts a potent anti-inflammatory effect on the brain [32]. In support of this, decreased cerebral NE levels and exacerbated neuroinflammatory processes have been demonstrated in murine AD models [27, 33]. Moreover, it is suggested that the reduction of LC noradrenergic neurons in the brains of AD patients could be one factor responsible for the exaggerated neuroinflammation in the brains of these patients [34]. Based on these facts and assumptions, the question has been raised as to whether the reduction of brain’s tissue NE levels in transgenic (SHR72) rats is accompanied by altered immune status in the brain of these animals. Therefore, we analyzed the gene expression of IL-6, one of the main immune system factors in the brain [35]. In transgenic rats, we found a significant elevation of IL-6 mRNA in CA, DG, and FCx. Moreover, significantly elevated IL-6 mRNA levels were also found in the brainstem, particularly in the LC, A1, and A2 noradrenergic cell groups. Increased gene expression of IL-6 in forebrain structures may reflect the reduced anti-inflammatory effect of NE due to reduced NE concentrations in these forebrain areas, while the increased IL-6 mRNA levels in brainstem noradrenergic areas may result from the burden of tau pathology, as it is localized mainly in the brainstem of transgenic (SHR72) rats [16].

It is suggested that stress represents important factor participating in the development of AD (for review, see [12]). Moreover, AD-related neuropathology may affect the stress response as well [36]. Because the LC represents a key structure modulating stress responses in the brain [23] and that we found that tauopathy impairs LC function, we investigated the effect of single and repeated stress on tissue levels of NE against the background of tauopathy. Interestingly, the exposure of transgenic rats to stress did not significantly affect NE levels when compared to unstressed transgenic animals. However, in the CA, DG, FCx, and NBM, NE levels in transgenic animals were significantly lower when compared to the NE levels of corresponding wild-type groups. These findings indicate that tauopathy reduces noradrenergic neurotransmission not only at basal conditions but also in animals exposed to stressors. Furthermore, these reduced NE levels in the forebrain areas of stressed, transgenic rats were accompanied by elevated gene expression of IL-6 in the CA and DG. Moreover, IL-6 mRNA levels were also elevated in brainstem A1 noradrenergic cells of stressed, transgenic animals. These findings indicate that the immune milieu remains altered in investigated brain areas in stressed, transgenic rats, but stress is not able to significantly affect gene expression of IL-6. It can be speculated that exposure to immobilization stress daily for 7 days is not sufficient to alter the immune status of transgenic rats and therefore longer period of chronic stress exposure will be necessary to determine the effect of stress on neuroinflammation at the background of tauopathy.

In transgenic (SHR72) rats, tau pathology is predominantly found in the brainstem [16]. However, even if reduced NE levels in forebrain areas of transgenic rats indicate functional impairment of the LC, we did not find any neurodegenerative changes at the level of the LC morphology (unpublished data). Using immunohistochemistry, we found accumulation of tau pathology in the form of AT8-immunopositive tau, pre-tangles, and neurofibrillary tangles in brainstem areas that innervate the LC, particularly in the paragigantocellular nucleus and hypoglossal preposite nucleus (unpublished data). Neurons of the paragigantocellular nucleus project to the LC and increase noradrenergic neurotransmission in many forebrain areas, including the hippocampus, via mostly glutamatergic excitatory fibers [37]. Therefore, we speculate that in transgenic (SHR72) rats, the tauopathy found in the paragigantocellular nucleus attenuates glutamatergic neurotransmission from the LC, which consequently reduces levels of NE in forebrain structures. However, alternative mechanisms may participate in the impairment of the LC as well, including reduced axonal transport of trophic factors to the nuclei of LC neurons from structures innervated by the LC.

To further investigate the functional impairment of the LC, we determined gene expression and protein levels of TH, the rate-limiting enzyme of NE biosynthesis as TH mRNA levels reflect changes in activity of the noradrenergic cells in this brain area [23]. We detected a significant increase of TH mRNA in the LC of repeatedly stressed wild-type animals that is in accordance with our previous reports [23, 38–41]. Importantly, TH protein concentration in the LC is in good agreement with TH expression. When compared to wild-type rats, transgenic (SHR72) animals started from the higher basal levels of TH mRNA, but did not show any significant changes after stress exposure. Increased TH levels in transgenic rats indicate exaggerated NE biosynthesis. However, as discussed above, NE levels in forebrain areas innervated by the LC were reduced. There is published data showing that even if the number of LC neurons in the brain of AD patients is significantly reduced, the remaining LC neurons undergo significant compensatory changes [42]. Our contradictory findings indicate that even if compensatory mechanisms in noradrenergic neurotransmission machinery are activated at the level of the LC, these responses are not able to compensate for the functional deficits manifested by reduced NE concentrations in brain areas innervated by the LC.

Besides the LC, we also analyzed TH mRNA in other noradrenergic cell groups that participate in noradrenergic innervation of forebrain structures, particularly in the A1 and A2 noradrenergic cell groups. However, in contrast to the LC, the A1 and A2 noradrenergic cell groups predominantly innervate the hypothalamus [23]. Therefore, sparse information is available as to how neurofibrillary pathology affects these cell groups. We found that TH mRNA levels in wild-type animals were significantly elevated in A1 and A2 noradrenergic cell groups after repeated stress. Also, while TH mRNA in A1 noradrenergic cells group of transgenic rats started from elevated basal levels, no further changes were detected after either single or repeated stress. We suggest that similarly to LC, increased TH expression in the A1 noradrenergic cell group might represent a compensatory response to impairment of TH-positive A1 neurons in this transgenic (SHR72) animal model of tauopathy. However, AD-related neuropathology of A1 noradrenergic neurons has not yet been described in available literature. However, we found that the A2 noradrenergic cell group shows an increase of TH mRNA levels in repeatedly stressed wild-type animals not seen in transgenic rats. Because the A1 and A2 noradrenergic cell groups innervate mainly the hypothalamic nuclei, we also investigated changes in NE level in the hypothalamic paraventricular nucleus. Importantly, in contrast to significantly reduced NE levels in forebrain structures innervated by the LC, we found that NE levels were only non-significantly reduced in the hypothalamic paraventricular nucleus in transgenic rats (9.93 ± 0.94 ng/mg protein) compared to wild-type animals (16.38 ± 2.57 ng/mg protein). These findings indicate that tauopathy in transgenic (SHR72) rats affects predominantly noradrenergic neurotransmission in the hippocampus, nucleus basalis of Meynert, and association cortices, all of which are areas innervated by the LC and affected in AD patients.

To further to determine the mechanisms responsible for tau-related alteration of the LC in transgenic (SHR72) rats, we measured tissue mRNA levels of both pro- and anti-inflammatory factors, including TNF-α, iNOS2, IL-10, and TGFβ1. We found that gene expression of these factors differs between wild-type and transgenic rats at both basal and stressed conditions, although TNF-α mRNA levels in unstressed, transgenic rats were only non-significantly increased. However, even if gene expression of TNF-α declined in repeatedly stressed transgenic rats compared to unstressed transgenic rats, TNF-α mRNA in rats exposed to repeated stress was significantly elevated compared to the matched, wild-type group. Gene expression of iNOS2, another pro-inflammatory factor, was significantly increased at basal conditions and findings related to gene expression of TNF-α and iNOS2 indicate a pro-inflammatory milieu in the LC tissue of transgenic rats. Moreover, gene expression of the anti-inflammatory cytokine IL-10 was increased in unstressed transgenic rats as well as in transgenic animals exposed to a single stressor, indicating activation of compensatory immune mechanisms at the level of the LC against a background of tauopathy. Comparison of NE, IL-6 mRNA, TNF-α mRNA, and IL-10 mRNA in the LC of transgenic rats indicate that the anti-inflammatory effect of NE in the LC is impaired. Because tauopathy is localized predominantly to the brainstem of transgenic (SHR72) rats, several local factors may promote an inflammatory milieu in the LC as well. Reduced gene expression of the neuroprotective factor TGFβ1 in repeatedly stressed transgenic rats highlights impairment of neuroprotective mechanisms in the LC. Based on the abovementioned findings, we suggest that tauopathy affects function of brainstem nuclei innervating the LC and alters the brainstem immune milieu. These alterations might participate in neuroinflammation and the reduced neuroprotection in the LC accompanied by reduced release of NE by LC neurons into the forebrain areas of transgenic (SHR72) rats. Reduced NE release might then participate in the development of neuroinflammation in forebrain structures innervated by the LC.

To investigate the role of the LC dysfunction in observed alteration of immune milieu in forebrain areas of transgenic rats, we assessed the correlation between NE and IL-6 mRNA levels in both wild-type and transgenic rats at basal and stress conditions. Importantly, we found that even if there was no correlation between NE and IL-6 mRNA levels at basal conditions, transgenic rats exhibit approximately threefold higher IL-6 mRNA levels. These findings indicate that the inhibitory effect of brain NE on neuroinflammation is severally impaired against a background of tauopathy. However, the mechanisms and pathways responsible for this phenomenon need further investigation.

It is necessary to note that all abovementioned parameters (e.g., interleukins) were investigated in brain tissues obtained by microdissection (punching) technique. However, this method does not allow to determine whether alterations are present in neurons, glia, or both types of cells. Therefore, for more complex view of neuropathological consequences of tau pathology on the function of the LC and on neuroinflammation, other approaches (e.g., immunohistochemistry) must be used.

## Conclusions

Our data have shown that tau pathology in the brains of transgenic (SHR72) rats alters central noradrenergic neurotransmission, particularly in brain’s structures innervated by noradrenergic neurons, under basal conditions as well as after an exposure to single or chronic stress. We observed increases in IL-6 gene expression in brain structures that may reflect a reduced anti-inflammatory effectiveness of the LC noradrenergic system. Importantly, some in vitro and in vivo studies have provided evidence that increases in NE levels can attenuate AD-related neuropathology and neuroinflammation, while enhancing cognition [43, 44]. In support of this, some clinical studies have already confirmed that pharmacotherapy potentiating noradrenergic neurotransmission is able to improve cognitive symptoms, while reducing depression and aggression in AD patients [45–47].

However, it is necessary to note that it remains unclear whether tauopathy in transgenic (SHR72) rats affect LC neurons directly or indirectly. Indirect mechanisms of altered LC function in transgenic rats may include (i) impaired transport of trophic factors from brainstem structures affected by tauopathy to the LC and (ii) reduced synthesis of norepinephrine in varicosities of the LC neurons as a result of either the pro-inflammatory milieu or other mechanisms. Moreover, tauopathy in transgenic (SHR72) rats may also potentially affect other catecholaminergic cell groups, exaggerating the noradrenergic deficit followed by impairment of the brain milieu and development of neuropathology. However, it is unclear whether other monoaminergic cell groups are affected in this transgenic model, and therefore, this needs further investigation.

Based on our data, we suggest that transgenic rats of this study can be efficiently employed in detailed investigation of molecular mechanisms of neuronal degeneration related to tauopathy, neuroinflammation, and, more importantly, in the development of disease modifying strategy for treatment of Alzheimer’s disease and other tauopathies.

## Abbreviations

AD: Alzheimer’s disease
CA: cornu ammonis
DG: dentate gyrus
FCx: frontal cortex
IL-10: interleukin 10
IL-6: interleukin 6
IMO: immobilization
iNOS2: inducible nitric oxide synthases 2
LC: locus coeruleus
NBM: nucleus basalis of the Meynert
NE: norepinephrine
TCx: temporal cortex
TGFβ1: transforming growth factor beta 1
TH: tyrosine hydroxylase
TNF-α: tumor necrosis factor alpha
